# Do multiple physiological OCT biomarkers indicate age-related decline in rod mitochondrial function in C57BL/6J mice?

**DOI:** 10.3389/fnins.2023.1280453

**Published:** 2023-11-17

**Authors:** Cole Goodman, Robert H. Podolsky, Karen Lins Childers, Robin Roberts, Ryan Katz, Rida Waseem, Anuhya Paruchuri, Josh Stanek, Bruce A. Berkowitz

**Affiliations:** ^1^Department of Ophthalmology, Visual and Anatomical Sciences, Wayne State University School of Medicine, Detroit, MI, United States; ^2^Biostatistics and Study Methodology, Children’s National Hospital, Silver Spring, MD, United States; ^3^Beaumont Research Institute, Beaumont Health, Royal Oak, MI, United States

**Keywords:** photoreceptor, OCT, biomarker, mitochondria, energy, aging, plasticity

## Abstract

**Purpose:**

To test the hypothesis that rod photoreceptor mitochondria function *in vivo* progressively declines over time.

**Methods:**

2, 12, and 24 month-old dark- and light-adapted C57BL/6J (B6J) mice were examined by OCT. We measured (i) an index of mitochondrial configuration within photoreceptors measured from the profile shape aspect ratio (MCP/AR) of the hyperreflective band posterior to the external limiting membrane (ELM), (ii) a proxy for energy-dependent pH-triggered water removal, the thickness of the ELM-retinal pigment epithelium (ELM-RPE), and its correlate (iii) the hyporeflective band (HB) signal intensity at the photoreceptor tips. Visual performance was assessed by optokinetic tracking.

**Results:**

In 2 and 24 month-old mice, MCP/AR in both inferior and superior retina was smaller in light than in dark; no dark–light differences were noted in 12 month-old mice. Dark-adapted inferior and superior, and light-adapted superior, ELM-RPE thickness increased with age. The dark–light difference in ELM-RPE thickness remained constant across all ages. All ages showed a decreased HB signal intensity magnitude in dark relative to light. In 12 month-old mice, the dark–light difference in HB magnitude was greater than in younger and older mice. Anatomically, outer nuclear layer thickness decreased with age. Visual performance indices were reduced at 24 month-old compared to 2 month-old mice.

**Conclusion:**

While the working hypothesis was not supported herein, the results raise the possibility of a mid-life adaptation in rod mitochondrial function during healthy aging in B6J mice based on OCT biomarkers, a plasticity that occurred prior to declines in visual performance.

## 1 Introduction

It is commonly hypothesized that during normal aging, mitochondria function declines, especially in cells such as rod photoreceptors, which are among the most metabolically active in the body (Bratic and Larsson, 2013; Haas, 2019; Lima et al., 2022). A decreased mitochondrial energy output may increase susceptibility of rods to energy substrate deficits and subsequent morbidities (Bratic and Trifunovic, 2010; Ingram et al., 2020). However, support for the above hypothesis is typically based on data from cell culture, *ex vivo* tissue samples, or drug-response studies, and support for this hypothesis *in vivo* is minimal (Barron et al., 2001; Wang et al., 2010; Alam et al., 2022). For example, electron microscopy and cytochrome C oxidase subunit 3 (COX III) measurements find increased mitochondria fragmentation and decreased COX III in 12 vs. 2 month-old mice; however, given that mice typically live much longer, 12 month-old does not represent advanced age (Kam and Jeffery, 2015; Hook et al., 2018). Separate studies have shown visual performance declines in the same mouse strain starting after 18 month-old of age and treatment with a mitochondrial therapeutic can mitigate these changes (Lehmann et al., 2012; Berkowitz et al., 2014, 2021; Alam et al., 2022). In older humans, a decrease in retinal mitochondrial metabolism has been suggested based on the level of cytochrome C complex oxidation, measured with broadband near-infrared spectroscopy (bNIRS); but this technique has a low spatial resolution, preventing differentiation between mitochondria in photoreceptors and other retinal layers (Kaynezhad et al., 2023). In addition, how optical media clarity anterior to the retina (e.g., lens opacity) modify the bNIRS signal is unknown. In summary, it remains unclear how rod mitochondria activity changes with age *in vivo*.

To begin to address this problem, we measured three OCT-based biomarkers of rod bioenergy (Berkowitz et al., 2022a,b). First, we measured the Mitochondria Configuration within Photoreceptors based on the profile shape Aspect Ratio (MCP/AR) of the hyperreflective band immediately posterior to the external limiting membrane (ELM). The MCP/AR index reflects mitochondria distribution within the photoreceptors inner segment, a region that is approximately 75% mitochondria by volume (Hoang et al., 2002; Spaide and Curcio, 2011). OCT imaging reveals that in C57BL/6 J (B6J) mice, MCP/AR increases in the dark (a high energy demand condition that supports the dark current) and decreases in the light (low energy demand) in agreement with electron microscopy measurements of mitochondria distribution (Berkowitz et al., 2022a). At this point, the mechanism(s) by which mitochondrial configuration within photoreceptors changes under light and dark conditions requires further investigation. Second, we measured the thickness of the outer retina between the ELM and retinal pigment epithelium (ELM-RPE). ELM-RPE thickness is driven by changes in pH linked to rod energy demand: high metabolic activity prompts pH-sensitive water removal co-transporters on apical RPE to dehydrate the subretinal space causing contraction (Li et al., 1994; Adijanto et al., 2009; Bissig and Berkowitz, 2012; Berkowitz et al., 2015; Li et al., 2016). Oxygen consumption rate measurements support the above interpretation of MCP/AR and ELM-RPE (Berkowitz et al., 2022a,b). Previous results are consistent with the notion that changes in ELM-RPE thickness are likely downstream from changes in MCP/AR with little evidence that they are simply linked via water movement (Berkowitz et al., 2022a,b). Lastly, we assessed the intensity of the hyporeflective band (HB) at the photoreceptor tips, measured as the absolute value of the peak reflectance amplitude from the baseline drawn between the photoreceptor tips and the RPE layer (Gao et al., 2021). The underlying mechanisms regulating dark–light changes in HB intensity are not clear at this point but we note that it has been correlated with ELM-RPE alterations as well as distribution changes in F-actin microfilaments in the cytoskeleton (Berkowitz and Qian, 2021; Gao et al., 2021; Berkowitz et al., 2022b). These findings are in-line with reports that water contributes to cytoskeleton dynamics (Lenormand et al., 2011).

In this study, we stress the need for all three OCT biomarkers to change in ways that agree with one another in order to reliably indicate low or high energy demand conditions. For example, with light adaptation, B6J mice showed a lower MCP/AR, thicker ELM-RPE, and greater HB intensity—evidence for lower retinal energy production—compared to 129S6/ev mice; the totality of these biomarker differences agree with results from a Seahorse assay (Berkowitz et al., 2022a). Similarly, B6J mice exposed to darkness display the distinct pattern of a relatively larger MCP/AR index, smaller ELM-RPE region, and minimal HB intensity compared to the biomarker pattern seen with lower energy demand in light. A concordant pattern of OCT biomarkers that agreed with measurements of oxygen consumption rate was also seen in rd17 mice (Berkowitz et al., 2022b). Such concordance is necessary because it can be challenging to interpret the meaning of a change in any particular imaging biomarker. In summary, agreement among all three biomarkers provides confidence in interpreting energy changes; if the three biomarkers do not change in concert, then interpretations that are not necessarily linked to mitochondria activity can be considered.

In this study, we tested a commonly proposed hypothesis that mitochondrial activity progressively decreases with age by testing whether changes in all three OCT biomarkers between 2, 12, and 24 month-old mice reflect a steady decline in mitochondria activity. Should this hypothesis hold true, we anticipated observing progressive reductions in MCP/AR, increased ELM-RPE thickness and HB intensity, and a gradual decline in dark–light differences as the mice aged. Companion anatomical and visual performance indices were also evaluated.

## 2 Methods

### 2.1 Animals

This study strictly adhered to the guidelines of the National Institutes of Health Guide for the Care and Use of Laboratory Animals, the Association for Research in Vision and Ophthalmology Statement for the Use of Animals in Ophthalmic and Vision Research, and was specifically authorized by the Wayne State University Division of Laboratory Animal Resources Institutional Animal and Care Use Committee (IACUC). We used male C57BL/6 J mice at three different age stages (2, 12, and 24 month-old) supplied by Jackson Laboratories (Bar Harbor, ME, United States). All mice lived under laboratory conditions that included a 12-h dark–light cycle. After data collection, euthanasia was performed humanely using an overdose of ketamine/xylazine, followed by cervical dislocation, according to our IACUC-approved protocol. Data were collected from the left eye of each mouse (B6J; 2 and 12 month-old: *n* = 10 dark, *n* = 10 light per age group, 24 month-old: *n* = 9 dark, *n* = 8 light).

### 2.2 OCT

**Figure 1:**
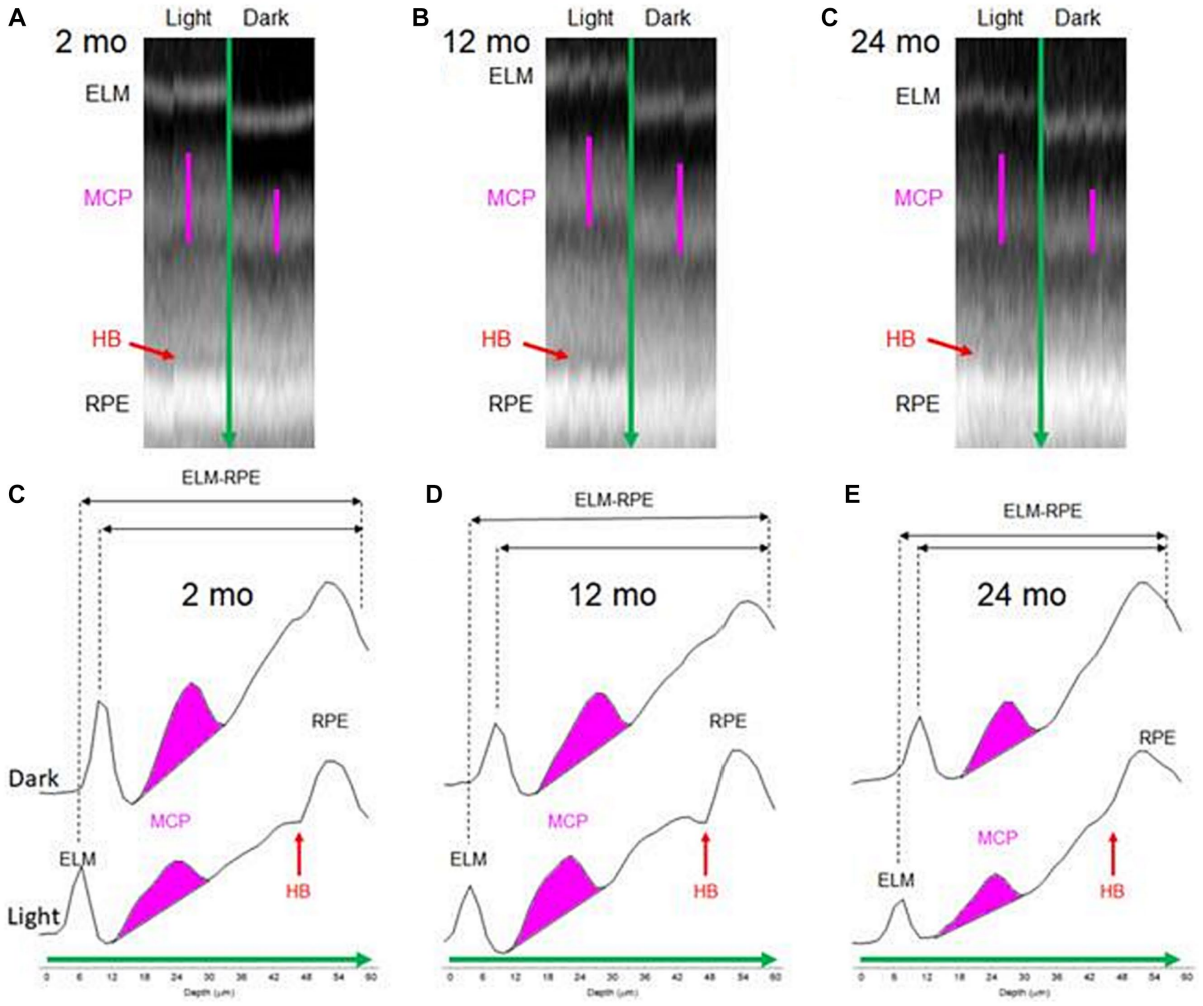
Representative data of outer retina in 2, 12, and 24 month-old mice shown as OCT B-scans **(A–C)** and corresponding A-line (reflectivity) profiles **(D–F)**. In **(D–F**), bottom profiles were collected from light adapted conditions and top profiles were collected from dark adapted conditions. *Green arrow*: direction of A-line profiles in **(D–F)**. *Pink line in **(A–C)***: Region of interest corresponding to MCP/AR. *Red arrow in **(A)***: Hyporeflective band between the photoreceptor tips and apical RPE. **(D–F)** The regions from MCP (*pink*), ELM-RPE (*black*), and HB (*red*) indexes are indicated. HB intensity is small in dark conditions (not depicted).

Using a cross-sectional design, mice at various ages were either adapted to room light for an hour after dark adaptation overnight, or kept in the dark for data collection. All imaging was completed in the morning (i.e., before noon) using an Envisu UHR2210 OCT (Bioptigen, Inc., Morrisville, NC, United States). Mice were anesthetized using a cocktail of 100 mg/kg ketamine and 6 mg/kg xylazine (Sigma-Aldrich, St. Louis, MO, United States). After their pupils were dilated with 1% atropine sulfate and eyes lubricated with Systane Ultra, a detailed scan of the central retina was obtained via radial volume scans. The radial volume scan parameters were: A-scans/B-scans = 1,000 lines; B-scans/volume = 1,000 scans; Frames/B-scan = 1 frame. A collection of 100 images extracted from B scan numbers 450–549, corresponding to the inferior–superior retina, were registered using an in-house developed R script. The initial rigid body registration step involved the RNiftyReg function in R to perform image rotation and signal interpolation at each pixel, followed by non-rotational rigid-body processes applied thrice at pixel row or column level. These 100 images were averaged. Separate analyses were carried out for inferior and superior retina areas (±350 to 624 μm from the optic nerve head). The intensity values used for the MCP/AR index and HB index were sourced from a log-based image (default in the Bioptigen system). In essence, ImageJ’s built-in functions were used to characterize the MCP/AR after fitting the profile shape of the hyperreflective band immediately posterior to the ELM to an ellipse matching the area, orientation, and centroid. From this ellipse, we derived a minor/major aspect ratio used as an unbiased, single value outcome index (i.e., the MCP/AR). Two other biomarkers—ELM-RPE thickness and the hyporeflective band signal intensity—were measured using our in-house R scripts which extracted layer boundaries, as previously described (Berkowitz et al., 2022b, 2023). In short, to measure retinal layer thicknesses a U-net convolutional neural network was employed followed by post-processing using a shortest-path algorithm (Berkowitz et al., 2022b, 2023). An R script was then applied to segment the image and extract the target indices. The hyporeflective band signal intensity magnitude was calculated from an R script as previously described, by analyzing profile contours that spanned the RPE and outer segment tips: A line was drawn straight between the RPE and the profile’s outer segment tip sections (intersecting only one point on each side of the hyporeflective band), and the largest deviation from that line indicated the hyporeflective band signal intensity magnitude (Berkowitz et al., 2020; Gao et al., 2021). Figure 1 shows representative images for each age group, highlighting the regions-of-interest for all three biomarkers under both light and dark conditions.

### 2.3 Optokinetic tracking

Two measures of visual performance were obtained: spatial frequency thresholds [SFT, “acuity,” in cycles/deg. (c/d)] and contrast sensitivity, measured at the peak of the nominal curve [(0.06 cycles/deg), inverse Michelson contrast (unitless); OptoMotry; CerebralMechanics, Inc., Alberta, Canada; Prusky et al., 2004; Douglas et al., 2005; Berkowitz et al., 2014]. The testing procedure involved projecting a vertical sine wave grating onto monitors placed in a quadrangle within the cylindrical testing arena. The procedure involved positioning unrestrained mice onto a central elevated platform within the arena. An operator then tracked the head movements of each mouse through an overhead video display, utilizing a computer mouse and superimposed crosshair which was continually updated to track the animal’s head movements. This method allowed for the real-time adjustment of the X-Y positional coordinates of the crosshair, centralizing the hub of the virtual cylinder and thus standardizing the distance from the wall of the cylinder to the animal’s eyes. This step keeps the spatial frequency of the stimulus consistent at the mouse’s viewpoint. As the virtual cylinder was turned either clockwise or counterclockwise, the mouse’s ability to perceive the grating was assessed based on the corresponding head and neck movements that followed the cylinder’s rotation. Depending on the direction of the cylinder’s rotation, measures for spatial frequency thresholds (SFT) and contrast sensitivity (CS) were derived for the left and right eye (Douglas et al., 2005). After an initial light exposure period of 1 h, each mouse’s SFT and peak CS measures were measured within a half-hour time frame. Both OCT and optokinetic tracking (OKT) measurements were conducted before noon, with the OKT test occurring 1 day prior to OCT.

### 2.4 Statistical analysis

Results are presented as means with 95% confidence intervals. A significance level of 0.05 was used for all tests. For each mouse, we first averaged each layer thickness and MCP/AR measured ±350 to 624 μm from the optic nerve head. We used the same modeling strategy for all outcomes (MCP/AR, layer thickness, magnitude of the hyporeflective band signal intensity, and OKT). A generalized linear mixed model was used that included the fixed effects of light/dark (omitted for OKT since mice are only evaluated in light), side (inferior vs. superior), age (2, 12, and 24 month-old), and all interactions. A random intercept for mouse within age and light condition as well as a random intercept for batch (i.e., animal group) were included as random effects. All outcomes except contrast sensitivity used a normal distribution, with contrast sensitivity being modeled using a gamma distribution with a log link. We evaluated whether residual variances depended on light condition or age group using the Akaike and Schwarz Bayesian information criteria (AIC and BIC). The final model for HB included heterogeneity of the residual variance by light condition since the AIC and BIC decreased >10 relative to a model with homogeneous variances. None of the other outcomes showed a decrease in either AIC or BIC greater than 10 leading us to assume the residual variance was constant across both age group and condition. We used Kenward-Roger degrees of freedom in testing all fixed effects for these models. Interactions that were not significant were removed to obtain the final model for each outcome. The fixed effects included in the model for each outcome were as follows: contrast sensitivity and acuity-age group and side; MCP/AR and ELM-RPE thickness—age group, light condition, side and all interactions; HB—age group, light condition, side, the age group by light condition interaction and the light condition by side interaction; ONL—age group, light condition, side, and the age group by side interaction. These models were fit using Proc Mixed and Proc Glimmix in SAS/STAT v15.1 (© 2016).

## 3 Results

Figure 1 shows how all three biomarkers responded to light and dark conditions as a function of age in representative mice. The following sections summarize the statistical differences observed in each biomarker as they relate to age.

### 3.1 MCP/AR index in light and dark

**Figure 2:**
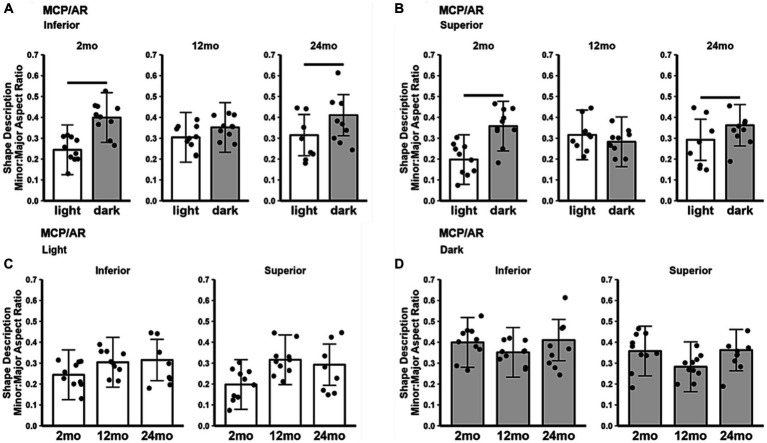
MCP/AR index in light and dark as a function of age. Summary of **(A)** inferior and **(B)** superior retina in light (white bars), and dark (gray bars) in 2, 12, and 24 month-old mice. **(C)** Comparison of light-only and **(D)** dark-only inferior and superior retina in each mouse group. Black horizontal line *p* < 0.05 (2-tailed, linear mixed model analysis; mean ± 95% CI; individual data points shown). In light: 2 month-old (*n* = 10), 12 month-old (*n* = 10), 24 month-old (*n* = 8); in dark: 2 month-old (*n* = 10), 12 month-old (*n* = 10), 24 month-old (*n* = 9).

In 2 and 24 month-old B6J mice, the MCP/AR indices in both inferior (Figure 2A) and superior (Figure 2B) retina were smaller in the light than in the dark, consistent with the lower photoreceptor energy demand in light conditions (Okawa et al., 2008; Ingram et al., 2020). On the other hand, 12 month-old B6J mice did not show a significant difference in dark–light MCP/AR index. Also, the MCP/AR index did not show statistically significant changes between age groups in either light only (Figure 2C) or dark only (Figure 2D) conditions.

### 3.2 ELM-RPE thickness in light and dark

**Figure 3:**
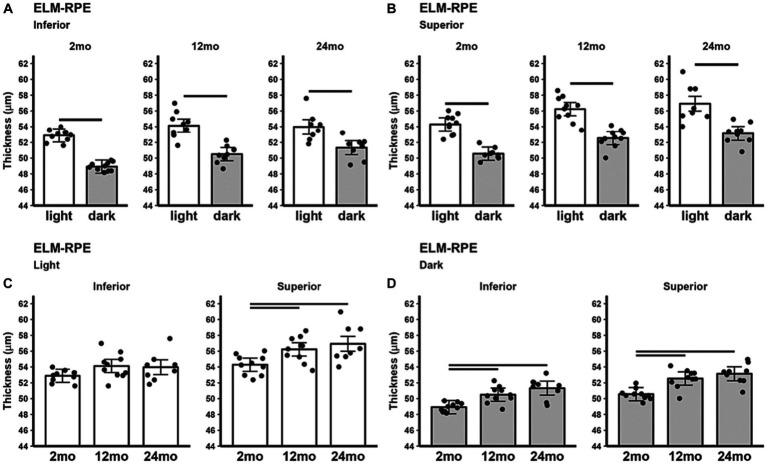
ELM-RPE thickness in light and dark as a function of age. Summary of **(A)** inferior and **(B)** superior retina light (white bars), and dark (gray bars) in 2, 12, and 24 month-old mice. **(C)** Comparison of light-only and **(D)** dark-only inferior and superior retina in each mouse group. Black horizontal line *p* < 0.05 (2-tailed, linear mixed model analysis; mean ± 95% CI; individual data points shown). Other details are listed in Figure 1.

The inferior (Figure 3A) and superior (Figure 3B) ELM-RPE were thicker in the light than in the dark, regardless of age. When light adapted, both 12 and 24 month-old B6J mice showed statistically significant thicker ELM-RPE than in 2 month-old B6J mice in superior retina; no such differences were noted in inferior retina (Figure 3C). When dark adapted, 12 and 24 month-old mice showed statistically significant thicker ELM-RPE than in 2 month-old mice in both inferior and superior retina (Figure 3D).

### 3.3 Hyporeflective band signal intensity in light and dark

**Figure 4:**
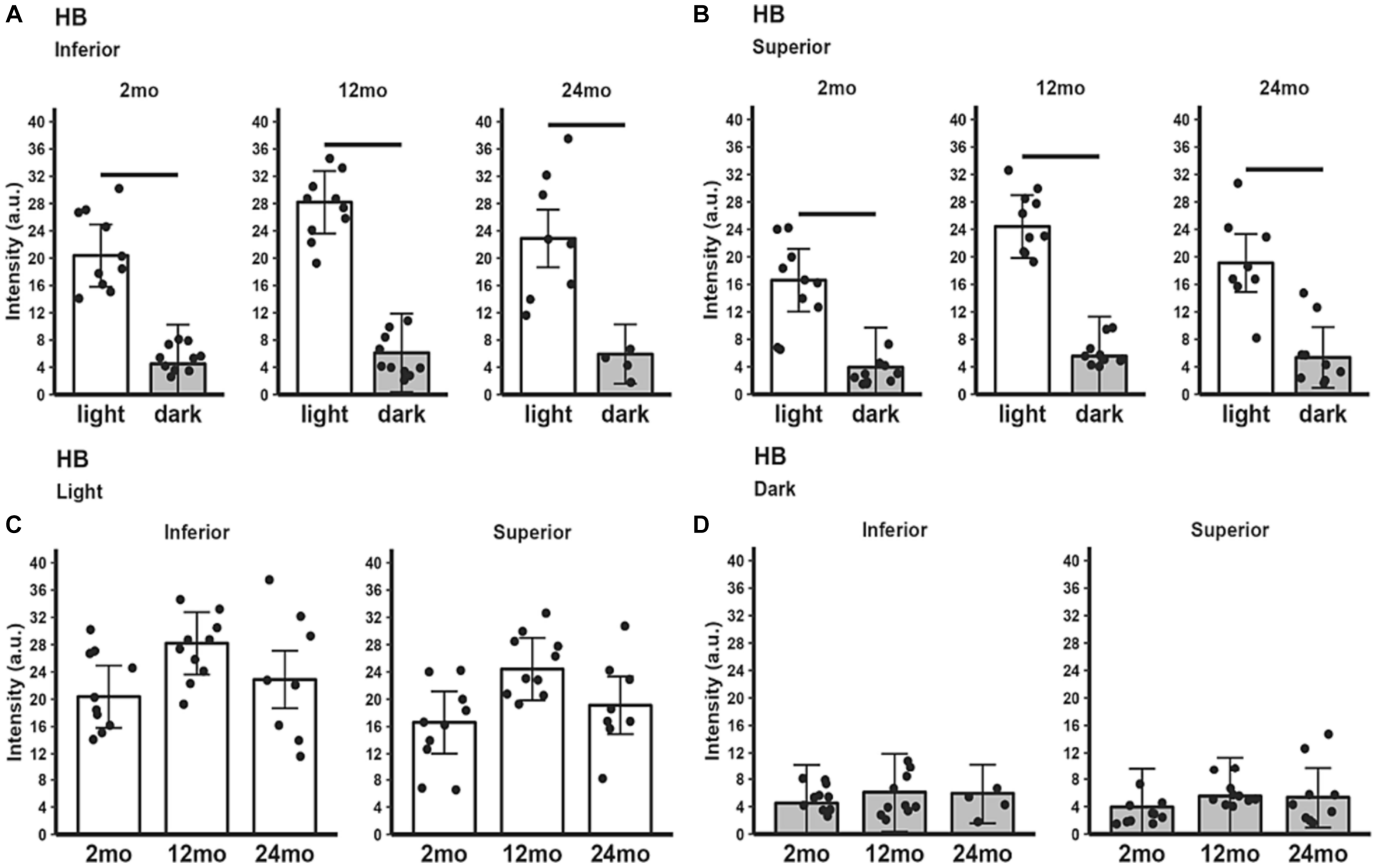
HB signal intensity in light and dark as a function of age. Summary of **(A)** inferior and **(B)** superior retina light (white bars), and dark (gray bars) in 2, 12, and 24 month-old mice. Comparison of **(C)** light-only and **(D)** dark-only inferior and superior retina in each mouse group. Black horizontal line *p* < 0.05 (2-tailed, linear mixed model analysis; mean ± 95% CI; individual data points shown). Other details are listed in Figure 1.

All three age groups showed an increased hyporeflective band signal magnitude in the light compared to the dark (Figures 4A,B). HB signal magnitude did not show statistically significant changes between age groups in either light only (Figure 4C) or dark only (Figure 4D) conditions.

### 3.4 Magnitude of dark–light differences in MCP/AR, ELM-RPE, and HB

**Figure 5:**
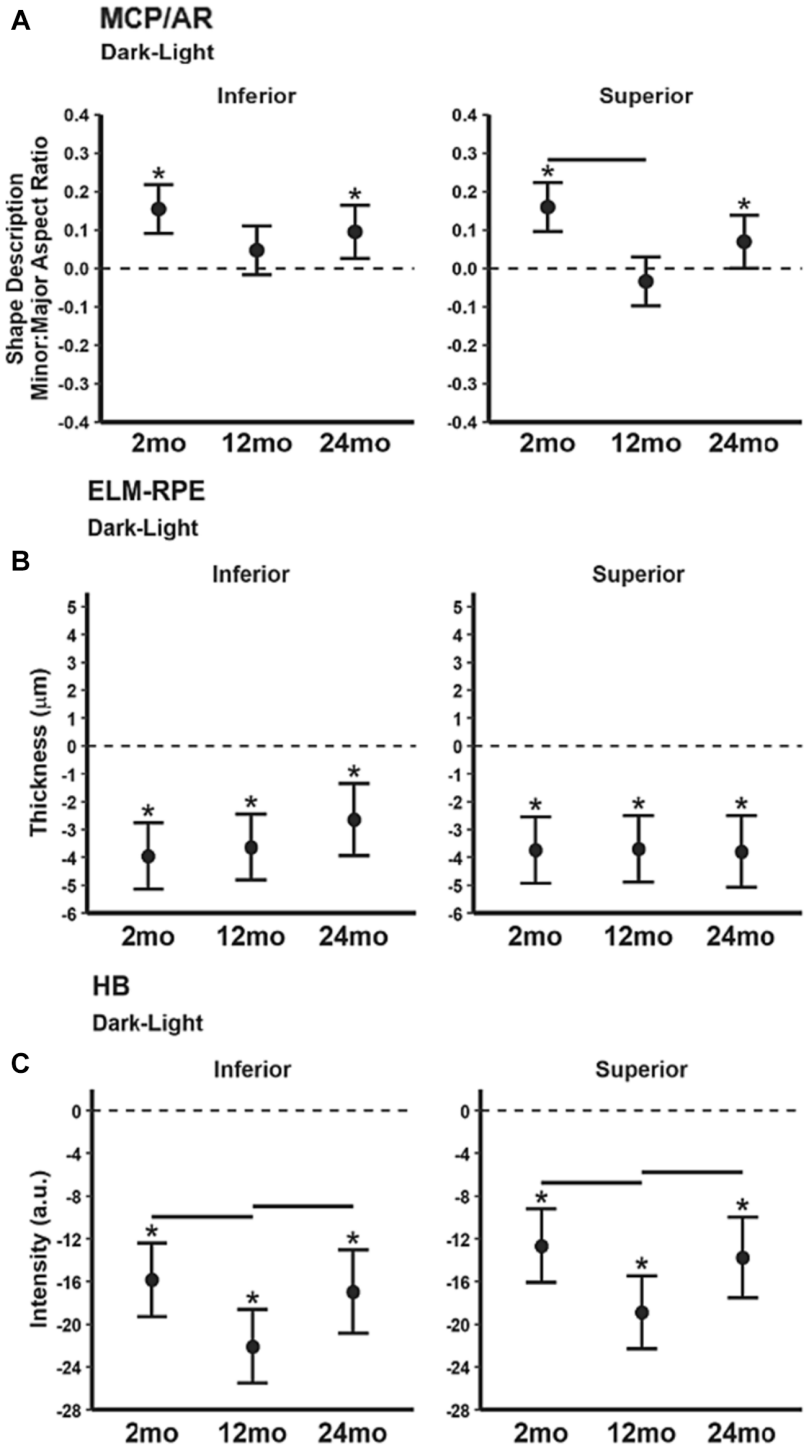
Summary of magnitude of dark–light differences as a function of age for each index **(A)** MCP/AR, **(B)** ELM-RPE, and **(C)** HB signal intensity. Black horizontal line *p* < 0.05 (2-tailed, linear mixed model analysis; mean ± 95% CI; individual data points shown). Stars indicate differences *p* < 0.05 (2-tailed, linear mixed model analysis) from 0 (dotted horizontal line). Other details are listed in Figure 1.

The MCP/AR dark–light difference (Figure 5A) decreased in superior retina at 12 month-old relative to 2 month-old but did not vary with age in inferior retina. ELM-RPE dark–light difference (Figure 5B) was of similar magnitude in all ages in both inferior and superior retina. The magnitude of the dark–light HB intensity difference (Figure 5C) in 12 month-old mice was significantly greater than that in younger 2 and older 24 month-old mice.

### 3.5 ONL thickness in light and dark

**Figure 6:**
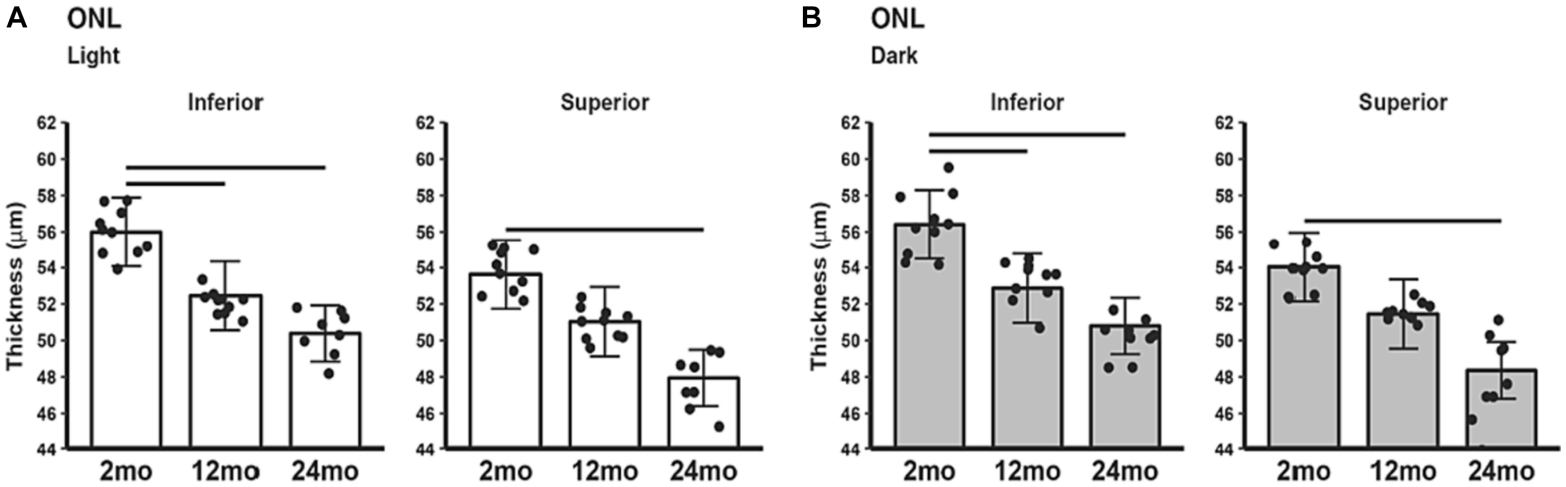
ONL thickness in light and dark as a function of age. Comparison of **(A)** light-only and **(B)** dark-only inferior and superior retina in 2, 12, and 24 month-old groups. Black horizontal line *p* < 0.05 (2-tailed, linear mixed model analysis; mean ± 95% CI; individual data points shown). Other conventions are listed in Figure 1.

The ONL thinned with age. In the light (Figure 6A) ONL thickness was decreased in 12 and 24 month-old B6J mice compared to 2 month-old mice in inferior retina and ONL thickness was decreased in 24 month-old, but not 12 month-old, compared to 2 month-old mice in superior retina. The same pattern was seen in the superior and inferior retina in dark adapted mice (Figure 6B). No differences in ONL thickness were noted between 12 and 24 month-old in light and in dark adapted mice.

### 3.6 Visual performance in light and dark

**Figure 7:**
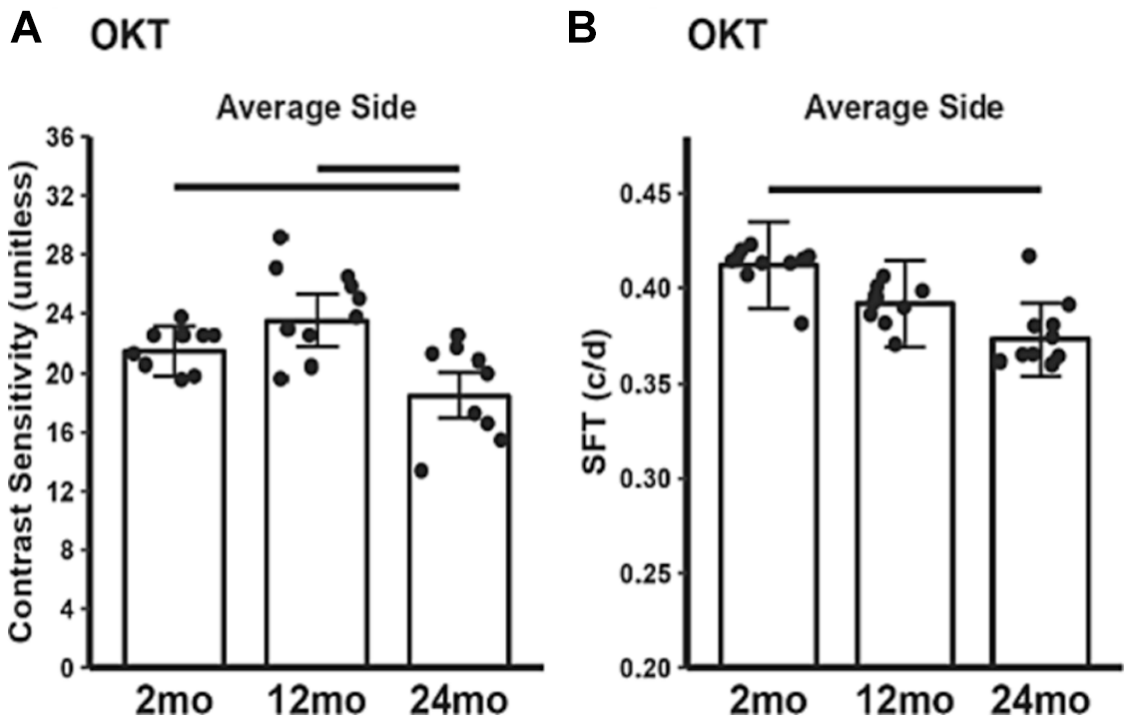
Summary of visual performance as a function of age measured with **(A)** contrast sensitivity and **(B)** spatial frequency threshold (SFT), a proxy for acuity. 2 month-old (*n* = 20), 12 month-old (*n* = 20), 24 month-old (*n* = 17). Black horizontal line *p* < 0.05 (2-tailed, linear mixed model analysis; mean ± 95% CI; individual data points shown).

Contrast sensitivity in 24 month-old mice was significantly reduced compared to both 2 and 12 month-old mice (Figure 7A). 24 month-old mice had lower visual acuity (SFT) than 2 month-old mice, but not 12 month-old mice (Figure 7B).

## 4 Discussion

In this study, ELM-RPE thickness increased with age, but this change did not coincide with associated changes in MCP/AR and HB intensity, with both MCP/AR and HB intensity remaining similar at all ages in light and dark adaptation. These results support the notion that these indices reflect different aspects of the outer retina energy ecosystem, in agreement with earlier findings (Berkowitz et al., 2022a,b). Thus, while the increased ELM-RPE thickness might be interpreted as a decline in mitochondrial activity with age, the lack of concordant changes in the other two biomarkers does not support this notion. Instead, the age-based increase in ELM-RPE thickness might reflect an alternative, non-mitochondrial mechanism. For example, ELM-RPE thickness is modulated by pH, and healthy aging is associated with acidosis in the brain (Decker et al., 2021). However, acidosis is expected to produce a contraction in the ELM-RPE region and not the expansion seen in the present study. Since it is unclear if acidosis also occurs during normal aging in B6J retina, further investigations into age-related pH changes in B6J rods are needed on this point.

We also considered alternative anatomical explanations for the above results. For example, might the increase in ELM-RPE thickness with age reflect an increase in outer segment length? Contrary to this idea, studies in B6J mice, other mouse strains, and rats suggest that rod outer segments do not lengthen with age (Trachimowicz et al., 1981; Fox and Rubinstein, 1989; Cunea and Jeffery, 2007; Kolesnikov et al., 2010). Further, if outer segment length did increase with age in B6J mice, one would expect an increase in energy-demanding cGMP channels. However, the overall changes observed in our biomarkers do not suggest an uptick in mitochondrial activity as they age. We also note that ONL thins with age. As previously discussed, this thinning is most consistent with retinal stretching in response to a steady growth of the globe and not rod degeneration in mice (Trachimowicz et al., 1981; Li et al., 2001; Samuel et al., 2011; Berkowitz et al., 2021; Ferdous et al., 2021). One consequence of this stretching could be a progressive reduction in the coverage and/or density of pH-responsive co-transporters in the RPE, leading to increased subretinal fluid retention and ELM-RPE thickness. An additional possible mechanism may involve accumulation of microglia in the subretinal space of aging mouse retina (Karlstetter et al., 2010; Ma and Wong, 2016; Guo et al., 2022). Overall, the OCT biomarker results, taken together, do not indicate a decrease in mitochondrial activity over time (i.e., only 1 of 3 biomarkers indicate a decreased energy output).

As summarized in Table 1, functional dark–light differences in mice across age groups were also measured. Again, concordant changes were not found, with distinct functional variations noted in two of the three biomarkers at 12 month-old but not at 24 month-old relative to 2 month-old mice. Specifically, in 12 month-old mice the MCP/AR dark–light difference decreased, and the HB dark–light difference increased. Overall, we find no consensus in the functional dark–light responses for the three biomarkers.

**Table 1:** Summary of evidence indicating mid-life plasticity in mitochondrial function (i.e., dark–light differences) as indicated by the lack of concordance among biomarkers.

Parameter	2 month-old	12 month-old	24 month-old
MCP/AR	+	−	+
ELM-RPE	+	+	+
HB	+	+(> than 2 and 24 month-old)	+

“+” = significant functional change measured; “−” = no significant functional change measured.

Next, we compared the present OCT biomarker findings with a report using more conventional assays. 12 month-old B6J mice were found to display increased mitochondrial fragmentation as measured by electron microscopy and a decrease in mitochondrial cytochrome C oxidase subunit 3 (COX III)—a critical regulatory protein of complex IV—as measured by immunohistochemistry of B6J eye isolates (Kam and Jeffery, 2015). Yet, that study did not evaluate visual performance or mitochondrial metabolism measured by, for example, retinal oxygen consumption rate. Furthermore, later time points, such as 24 month-old mice, were not interrogated, making the implications of the electron microscopy and COX III indices for aging unclear (Kam and Jeffery, 2015). We note that the present results are not inconsistent with an increased mitochondrial fragmentation and COX III decline at 12 month-old. Interestingly, despite these findings, we did not observe a decline in visual performance in 12 month-old B6J mice, an outcome one might anticipate if mitochondria were compromised (Lehmann et al., 2012; Berkowitz et al., 2014, 2021; Alam et al., 2022). In summary, the totality of the OCT biomarker changes noted herein do not appear related to a progressive decline in mitochondrial metabolism.

It is intriguing that functionally, dark–light ELM-RPE differences are preserved across all age groups, as we expected this difference to decline with loss of mitochondrial activity. Above, we considered the possible contribution of microglia to subretinal space thickness. If microglia did play a role in the dark–light ELM-RPE difference changes, we reason—on mechanical grounds—that this difference would likely decline over time as microglial quantity increases. However, this was not observed. Additional studies are needed to further understand how dark–light ELM-RPE differences are sustained over time.

It is noteworthy that our OCT biomarkers were similar in dark–light conditions for 2 and 24 month-old mice. This raises the possibility that instead of a progressive decline in mitochondria activity with age, compensatory mechanisms—that were evident by 12 month-old—maintained rod homeostasis over time. Indeed, evidence for a similar time course in plasticity in rod photoreceptor cells with age was also observed *in vivo* based on their L-type calcium channel (LTCC) activity, a linked signaling pathway regulating mitochondria activity (Hool and Viola, 2012; Berkowitz et al., 2014; Giorgi et al., 2018; Müller et al., 2018; Hotka et al., 2020). Further histochemical evidence for age-related plasticity in photoreceptors—namely sprouting of inner retina neurons into the ONL starting around 12 month-old of age in B6J mice—have also been presented (Liets et al., 2006; Zhu et al., 2023). Overall, based on the current OCT biomarkers, possible mid-life plasticity may contribute to a subsequent decline in visual performance. More studies are needed to examine this hypothesis.

This study has some limitations. The use of a single strain of male mice limits the generalizability of our findings and B6J mice lack the mitochondria nicotinamide nucleotide transhydrogenase (*Nnt*) gene which is thought to alter mitochondria regulatory processes and reduce ATP production efficiency (Ronchi et al., 2013). Additionally, while our biomarkers provide valuable insight, they are a proxy and do not measure mitochondrial function directly. Finally, we did not examine in this study the possibility that age-related media opacities contribute to reductions in contrast sensitivity and acuity. Previously we found no visible evidence on MRI for cataract although more subtle changes in media opacities have been suggested (Berkowitz et al., 2014; Ferdous et al., 2021). It seems unlikely that subtle changes in opacities are sufficient to cause the visual performance declines noted herein but further investigation is needed.

In summary, the results of this study did not support the hypothesis of a progressive age-related decline in photoreceptor mitochondrial function in B6J mice. Instead, they raise the possibility of a mid-life plasticity in rod mitochondria activity with possible implications for future visual impairment. As aging is the substrate on which many retinopathies develop, understanding how mitochondria change during healthy aging is key to comprehending the pathogenesis and treatment of vision-threatening retinal diseases.

## Data availability statement

The raw data supporting the conclusions of this article will be made available by the authors, without undue reservation.

## Ethics statement

The animal study was approved by Wayne State University Division of Laboratory Animal Resources Institutional Animal and Care Use Committee. The study was conducted in accordance with the local legislation and institutional requirements.

## Author contributions

CG: Investigation, Writing – original draft, Writing – review & editing, Visualization. RP: Formal analysis, Writing – review & editing, Project administration, Supervision, Visualization, Writing – original draft. KC: Formal analysis, Writing – review & editing, Visualization. RR: Investigation, Project administration, Resources, Writing – review & editing, Methodology, Supervision. RK: Investigation, Writing – review & editing. RW: Investigation, Writing – review & editing. AP: Investigation, Writing – review & editing. JS: Investigation, Writing – review & editing. BB: Funding acquisition, Investigation, Methodology, Resources, Writing – original draft, Writing – review & editing, Conceptualization, Data curation, Project administration, Supervision, Validation, Software, Visualization.
